# Development of an Internal Real-Time Wireless Diagnostic Tool for a Proton Exchange Membrane Fuel Cell

**DOI:** 10.3390/s18010213

**Published:** 2018-01-13

**Authors:** Chi-Yuan Lee, Chia-Hung Chen, Chao-Hsuan Tsai, Yu-Syuan Wang

**Affiliations:** 1 Department of Mechanical Engineering, Yuan Ze Fuel Cell Center, Yuan Ze University, Taoyuan 320, Taiwan; s1045017@mail.yzu.edu.tw (C.-H.T.); s1040935@mail.yzu.edu.tw (Y.-S.W.); 2 HOMYTECH Global CO., LTD., Taoyuan 33464, Taiwan; sv3@homytech.com

**Keywords:** PEMFC, MEMS, flexible integrated microsensor, real-time wireless diagnostic tool

## Abstract

To prolong the operating time of unmanned aerial vehicles which use proton exchange membrane fuel cells (PEMFC), the performance of PEMFC is the key. However, a long-term operation can make the Pt particles of the catalyst layer and the pollutants in the feedstock gas bond together (e.g., CO), so that the catalyst loses reaction activity. The performance decay and aging of PEMFC will be influenced by operating conditions, temperature, flow and CO concentration. Therefore, this study proposes the development of an internal real-time wireless diagnostic tool for PEMFC, and uses micro-electro-mechanical systems (MEMS) technology to develop a wireless and thin (<50 μm) flexible integrated (temperature, flow and CO) microsensor. The technical advantages are (1) compactness and three wireless measurement functions; (2) elastic measurement position and accurate embedding; (3) high accuracy and sensitivity and quick response; (4) real-time wireless monitoring of dynamic performance of PEMFC; (5) customized design and development. The flexible integrated microsensor is embedded in the PEMFC, three important physical quantities in the PEMFC, which are the temperature, flow and CO, can be measured simultaneously and instantly, so as to obtain the authentic and complete reaction in the PEMFC to enhance the performance of PEMFC and to prolong the service life.

## 1. Introduction

The crude oil price rose in recent years, and various countries actively developed new alternative energy sources, such as solar energy, wind power, hydrogen energy, hybrid electric vehicles and all electric vehicles, which are green energy. The generating set composed of hydrogen and oxygen becomes another usage mode of hydrogen energy. Its major advantages include high combustion heat value; the product of combustion is water, it is the cleanest energy in the world; abundant resources, the hydrogen can be made from water, and water is the most abundant resource of the earth. The proton exchange membrane fuel cell (PEMFC) is characterized by high power density, low operating temperature, quiet operation and lower corrosion, reduced laminated design, quick start and shutdown [1]. The reliability and durability of PEMFC have been mature, but there shall be good water and thermal management, and the purification and high cost problems of hydrogen shall be overcome. In terms of the fuel cell design and conception, high overall performance and long life of membrane electrode assembly (MEA) are required. The key factors influencing the fuel cell performance and life include the bipolar plates (BP), gas diffusion layer (GDL), catalyst layer (CL) and closing pressure. Generally, during the assembling of a fuel cell, the clearance between BP and MEA shall be sealed with high temperature glue or seal washer, so as to solve the gas leakage. 

Lim et al. [2] developed and designed a sort of BP, the sealing of fuel cell was free of high temperature glue or seal washer. 

Naing et al. [3] studied the effect of GDL on the fuel cell performance, and compared two GDLs in different directions to validate the water proof GDL and the delivery of fuel gas. 

The durability and performance of a catalyst are very important in PEMFC, the nano-powder of platinum and carbon is used for electrochemical reaction of fuel to produce water. The hydrogen of the anode reacts with the platinum catalyst; the hydrogen is decomposed into hydrogen ions and electrons. The hydrogen ions penetrate through the Proton Exchange Membrane (PEM) to the cathode, reacting with oxyanions to form water, and the electrons are transmitted by carbon paper to the BP to generate electric energy. Increasing the thermal stability property of CL and the utilization rate of platinum can result in higher current density [4]. 

Avcioglu et al. [5] studied the CL, and learned about the hydrophobic nanoparticles and electrochemical reaction in the CL. 

Goto et al. [6] developed a gold/indium oxide sensor, the minimum measurable CO concentration was 10 ppm, and the sensitivity was only affected by humidity, but this effect was almost negligible. 

Barroca et al. [7] developed an automatic wireless sensor system, composed of IEEE 802.15.4 network architecture and various commercial temperature and humidity sensors, put in concrete for wireless remote temperature and humidity monitoring. The results showed that the wireless sensor system could obtain the temperature and humidity information instantly and accurately.

Jung et al. [8] found that a wireless sensor network is emerging as an innovative method for gathering information that will significantly improve the reliability and efficiency of infrastructure systems.

Jiang et al. [9] developed a PdNi thin film hydrogen gas sensor with integrated Pt thin film temperature sensor was designed and fabricated using the micro-electro-mechanical systems (MEMS) process. 

Díaz-Álvarez et al. [10] found that thermal issues are critical when machining Ni-based superalloy components designed for high temperature applications. The low thermal conductivity and extreme strain hardening of this family of materials results in elevated temperatures around the cutting area. 

Wang et al. [11] presented a novel cluster-based maximum consensus time synchronization (CMTS) method. It consists of two parts: intra-cluster time synchronization and inter-cluster time synchronization.

Choi et al. [12] developed a minimum interference channel assignment (MICA) algorithm for multicast that accurately models the interference relationship between pairs of multicast tree nodes using the concept of the interference factor and assigns channels to tree nodes to minimize interference within the multicast tree.

In the past, our research team has successfully developed wired multi-function microsensors for vanadium redox flow battery [13], high temperature proton exchange membrane fuel cell [14] and lithium ion battery [15]. This paper proposes the development of internal real-time wireless diagnostic tool for PEMFC, and uses micro-electro-mechanical systems (MEMS) technology to develop a wireless and thin (<50 μm) flexible integrated (temperature, flow and CO) microsensor. 

## 2. Sensing Principle and INTEGRATION of Microsensors

### 2.1. Micro Temperature Sensor

**Figure 1:**
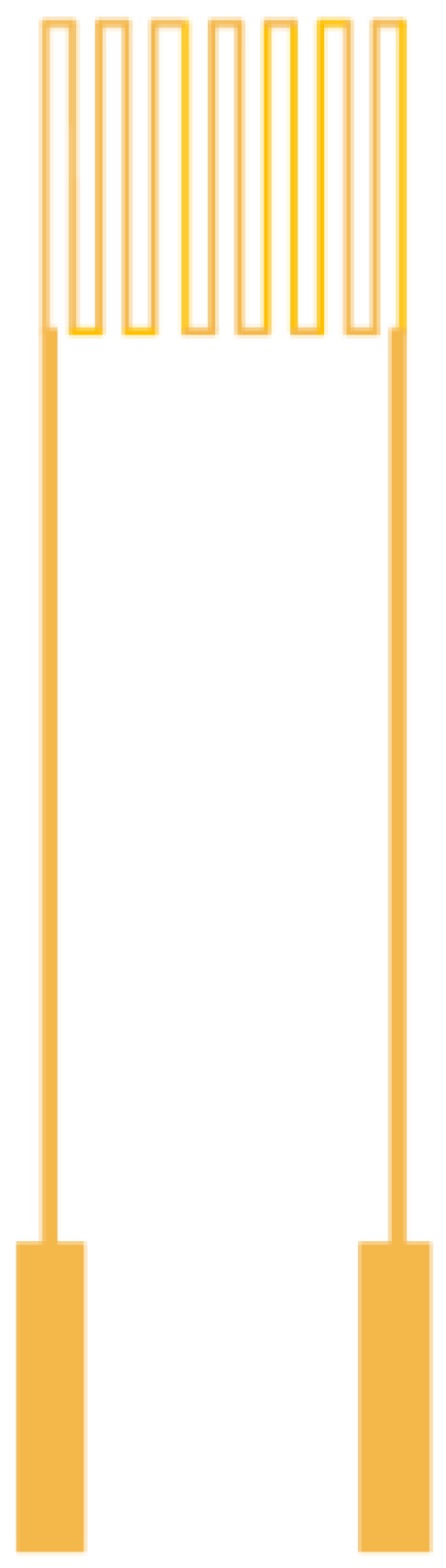
Schematic diagram of resistance temperature detector (RTD) structure.

The micro temperature sensor used in this study is resistance temperature detector (RTD) and the electrode type is serpentine, as shown in Figure 1. The RTD material is Au because of its stable chemical properties, simple process and high linearity.

The sensing principle of micro temperature sensor is that when the ambient temperature rises, as Au has a positive temperature coefficient (PTC), the resistivity of RTD increases. This characteristic results from the “Temperature Coefficient of Resistance” (TCR) of the conductor, defined as Equation (1).

(1) $$\alpha =\frac{1}{\rho_{0}}\frac{d\rho }{dT}$$

where α is the TCR; *ρ*_0_ is the resistivity at 0 °C.

Therefore, the relation between the resistivity of the conductor and temperature can be expressed as Equation (2).

*R_t_* = *R*_0_ (1 + *α*_1_ Δ*T* + *α*_2_ Δ*T*^2^ + *α*_3_ Δ*T*^3^ + …) (2)

Δ*T* = *t* − *t*_0_ (3)

where *R_t_* is the resistivity (Ω) at *t* °C; *R*_0_ is the resistivity (Ω) at 0 °C; *α*_1_, *α*_2_ and *α*_3_ are the TCR (%/°C); Δ*T* is the temperature difference (°C) in relation to reference temperature 0 °C; *t* is the temperature (°C) at *t* °C; *t*_0_ is the temperature (°C) at 0 °C.

Equation (2) shows that the relationship between the temperature and resistance value of conductor is nonlinear, but if the conductor resistance value of RTD is in the linear range, Equation (2) can be reduced to Equation (4).

*R_t_* = *R*_0_ (1 + *α*_1_ Δ*T*) (4)

### 2.2. Micro Flow Sensor

**Figure 2:**
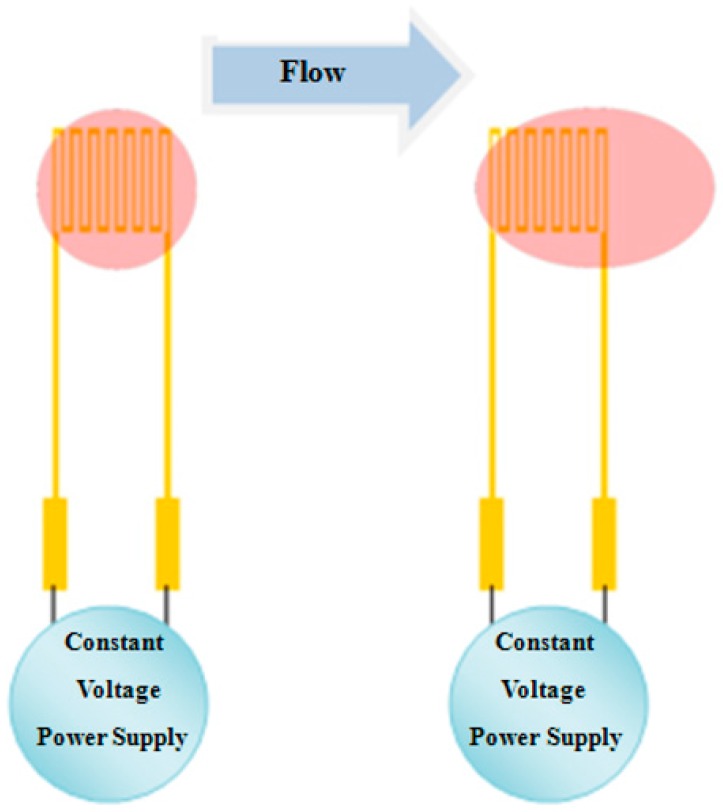
Schematic diagram of the principle of the hot-wire flow sensor.

The micro flow sensor used in this study is a hot-wire flow sensor, which is designed according to positive correlation between the thermal energy dissipation rate of hot wire and fluid flow. The schematic diagram of the hot-wire flow sensor principle is shown in Figure 2.

According to King’s law, the relationship between thermal energy dissipation rate and fluid flow rate is expressed as Equation (5).

*Q* = *I*^2^ × *R* = (*A* + *B* ×*U^n^*) (*T_s_* − *T_o_*) (5)

where *Q* is the electric power from the external power supply; *I* is the electric current through hot wire; *R* is the resistance of hot wire; *A* is the heat transfer coefficient of fluid; *U* is the flow rate of fluid; *n* is the coefficient of correlation between heat *Q* and flow rate *U*.

### 2.3. Micro CO Sensor

The micro CO sensor in this study is a semiconductor sensor. The principle is to use the reducing gas and the oxygen adsorption on the surface of gas-sensing thin film. When the oxygen atom (O) is adsorbed on the surface of gas-sensing thin film, it is likely to capture the electrons in the material to form adsorbates like oxyanion (O^−^). The electrons in the gas-sensing thin film are reduced, so that the resistance of the sensing material increases. When the surface of gas-sensing thin film comes into contact with the reducing gases (e.g., CO, H_2_) in the environment, the reducing gas reacts with the oxyanion adsorbed on the surface of gas-sensing thin film, performing an oxidation reaction with the oxyanion to release electrons into the gas-sensing thin film. The returned electrons reduce the overall resistance of the material.

The maximum adsorbance on the surface of gas-sensing thin film and the overall resistance of the thin film depend on the quantity of reducing gas, operating temperature of the gas-sensing thin film, quantity of adsorption sites and film surface activity in equilibrium state. The gas-sensing thin film used in this study is SnO_2_, which is sensitive to hydrocarbon compounds, and it has stable chemical properties and high production compatibility. It is the optimal gas-sensing material.

### 2.4. Integrated Design of Flexible Integrated Microsensor

**Figure 3:**
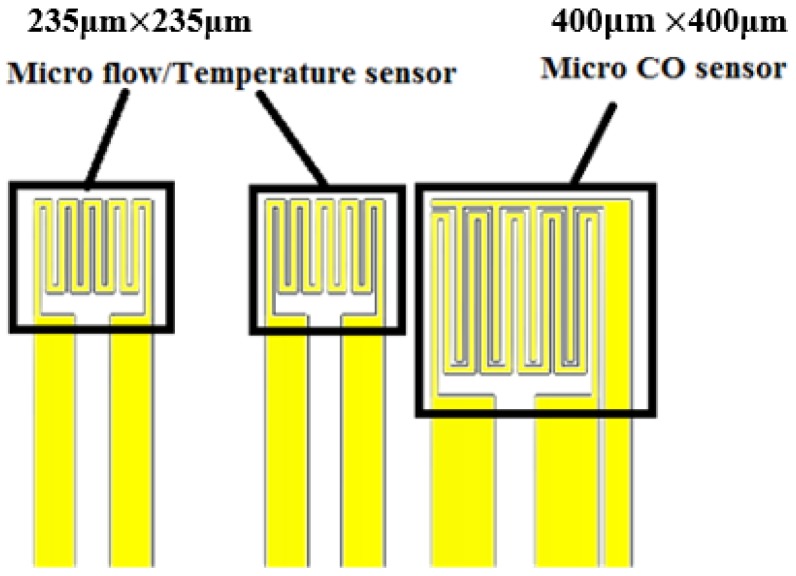
Design drawing of flexible integrated microsensor.

The flexible integrated microsensor design is shown in Figure 3 (temperature sensing area: 235 μm × 235 μm; flow sensing area: 235 μm × 235 μm; CO sensing area: 400 μm × 400 μm). This integrated design method not only reduces the coverage area of microsensor in the fuel cell, but also minimizes the effect on the overall performance of cell, and the temperature, flow and CO can be measured simultaneously.

## 3. Integrated Fuel Cell and Flexible Integrated Microsensor

After the flexible integrated microsensor is made and the PEMFC is established, the flexible integrated microsensor is packaged, the micro temperature, flow and CO sensors are corrected, and the reliability is validated. Three flexible integrated microsensors are embedded in the cathode channel plate of PEMFC in this study.

### 3.1. Flexible Integrated Microsensor and Fuel Cell Assembly

**Figure 4:**
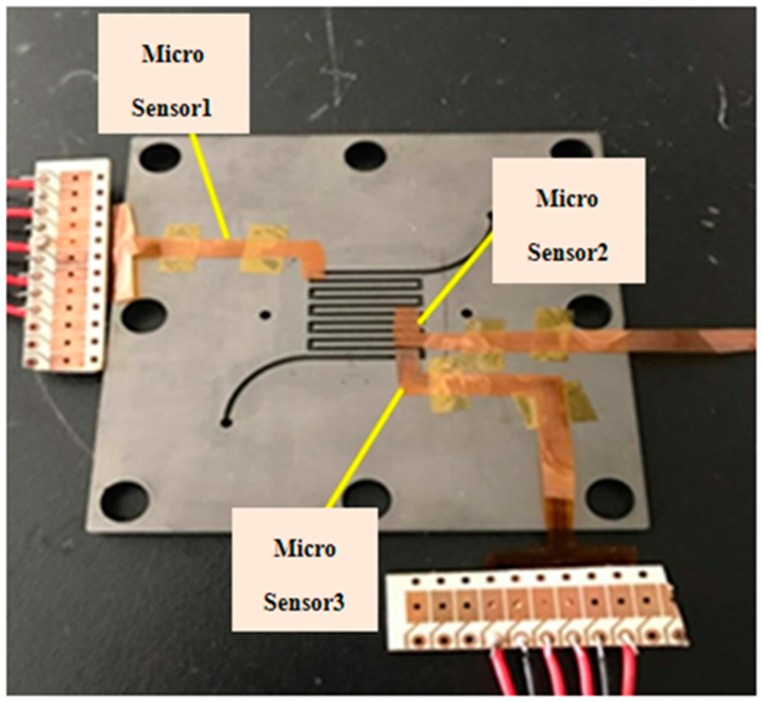
Schematic diagram of flexible integrated microsensor embedded in the cathode channel plate.

This study uses a 10 cm^2^ (reaction area: 6.25 cm^2^) fuel cell for testing. In order to stabilize the cell performance and to avoid the fuel gas leaking in the fuel cell testing process, the graphite bipolar plate breaks if the closing pressure is too high, and the gas leaks if the closing pressure is too low. This study uses closing pressure 25 kg/cm^2^ to close the cell uniformly. The flexible integrated microsensor is embedded above the rib of the cathode channel plate. The upper, middle and lower reaches are embedded with one sensor respectively, as shown in Figure 4.

### 3.2. Correction of Micro Temperature Sensor

**Figure 5:**
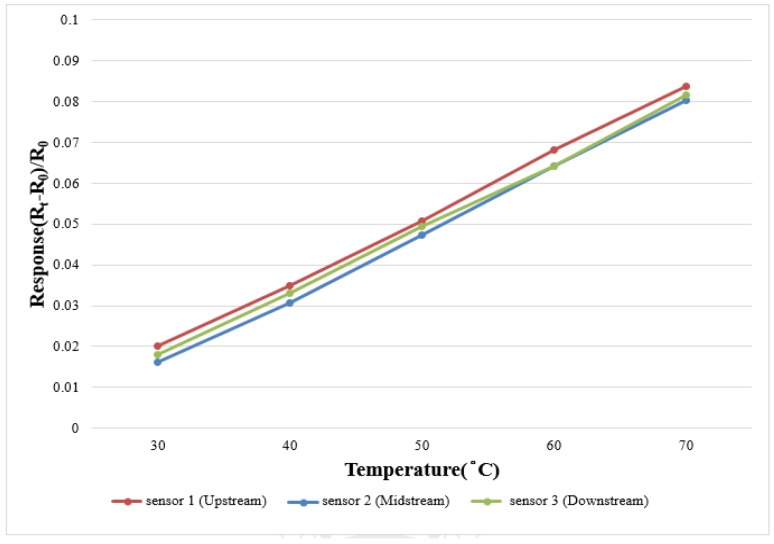
Temperature correction curve.

In order to make the correction environment closer to the practical situation, this study uses a program controlled constant temperature and humidity testing machine (Hung Ta HT-8045A Environmental Chamber) as the basis of correction environment. The data capture equipment for this study is NI PXI 2575 of National Instruments (NI) which can extract the resistivity of the micro temperature sensor instantly, and the LabVIEW system design software and control system are used for signal processing and analysis. Finally, the analyzed and processed data are exported to the computer to draw the correction curve. The temperature correction range of micro temperature sensor is 30 °C to 70 °C at intervals of 10 °C. Five signals are extracted, the correction is implemented three times and the average value is taken. The measured correction curve is shown in Figure 5. The results show that the micro temperature sensor has good linearity and reliability.

### 3.3. Correction of Micro Flow Sensor

**Figure 6:**
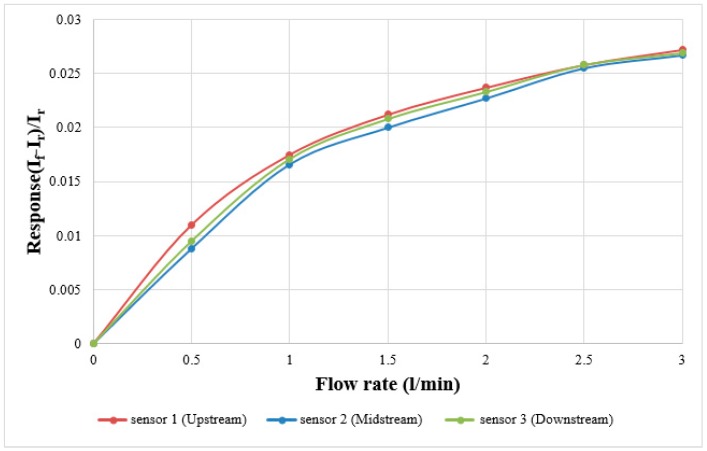
Flow calibration curve diagram.

In terms of flow calibration, the fuel cell testing machine supplies steady flow, the power supply and micro flow sensor are connected by a digital multimeter in series to measure the current variation value. The power supply generates a stable temperature field for the micro flow sensor by constant voltage, and the air is imported for correction. The flow correction range is 0 ~ 3 L/min, measured once at intervals of 0.5 L/min. The correction curve of the micro flow sensor is shown in Figure 6, where *I_f_* is the current value measured at flow *f*, *I_r_* is the current value of the flow reference point.

### 3.4. Correction of Micro CO Sensor

**Figure 7:**
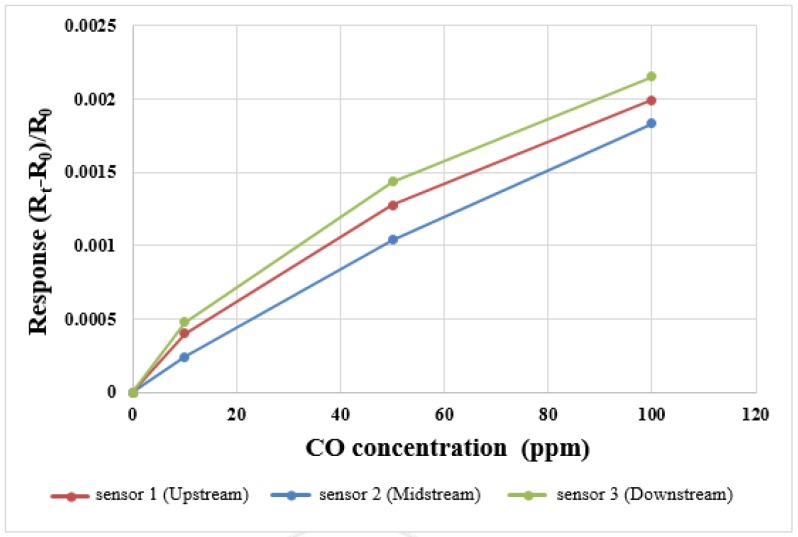
CO correction curve.

For the CO correction test, 10 ppm, 50 ppm and 100 ppm CO + N_2_ mixed gases were bought from Minyang Specialty Gases Co. Ltd. In Taiwan Taoyuan country, the input flow through the cathode terminal is 1.5 L/min, three points are corrected three times, the correction curve is shown in Figure 7.

## 4. Local Wireless Real-Time Microscopic Monitoring in Fuel Cell and Analysis

In the past, our research team has successfully developed wired multi-function microsensors for a vanadium redox flow battery, high temperature proton exchange membrane fuel cell and lithium ion battery. This paper proposes the development of an internal real-time wireless diagnostic tool for PEMFC, and uses MEMS technology to develop a wireless and thin (<50 μm) flexible integrated (temperature, flow and CO) microsensor. When the correction of a flexible wireless microsensor is completed, the fuel cell testing machine, NI wired and wireless data acquisition units are used for internal information acquisition and microscopic diagnostic analysis of a fuel cell. The performances of the cells with and without flexible integrated microsensor are compared, and the local temperature, flow and CO changes and distributions in the fuel cell are monitored and discussed under constant current, and the feasibility of wireless data acquisition unit is validated.

### 4.1. Fuel Cell Test Environment

**Figure 8:**
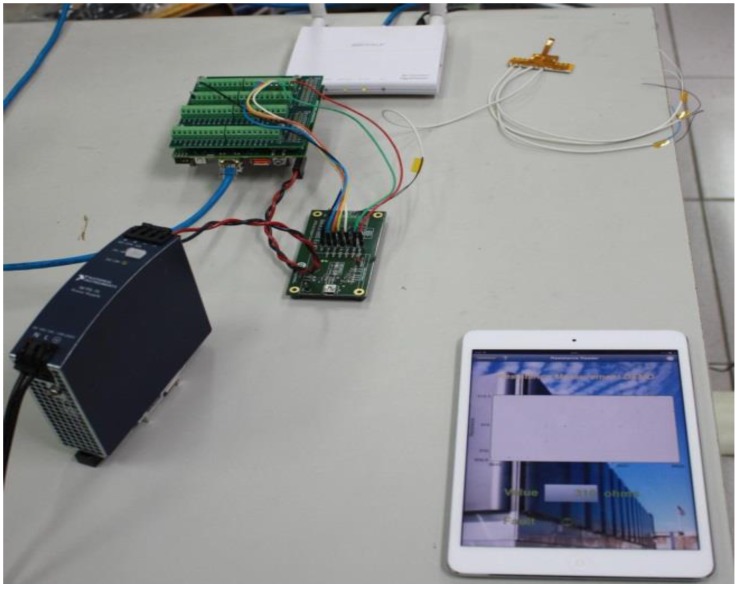
National Instruments (NI) wireless data module.

The fuel cell is tested by 100 W PEM Fuel Testing Station. The reaction area of a fuel cell is 6.25 cm^2^. Different unhumidified gas flows of the anode flow rate (H_2_, 0.5 slpm) and cathode flow rate (Air, 1.5 slpm) are given at an operating temperature of 50 °C. The performance curves of the fuel cells with and without a flexible integrated microsensor are measured respectively, and the difference is worked out, so as to give different loads constant current (2, 3.5, 6 A). The local physical quantities in the cell are obtained by NI PXI 2575 wired data acquisition unit and NI wireless data module (Figure 8), discussed and analyzed. The detailed operating conditions are compiled in Table 1.

### 4.2. Comparison between Performances of Cells with and without an Embedded Flexible Integrated Microsensor

**Figure 9:**
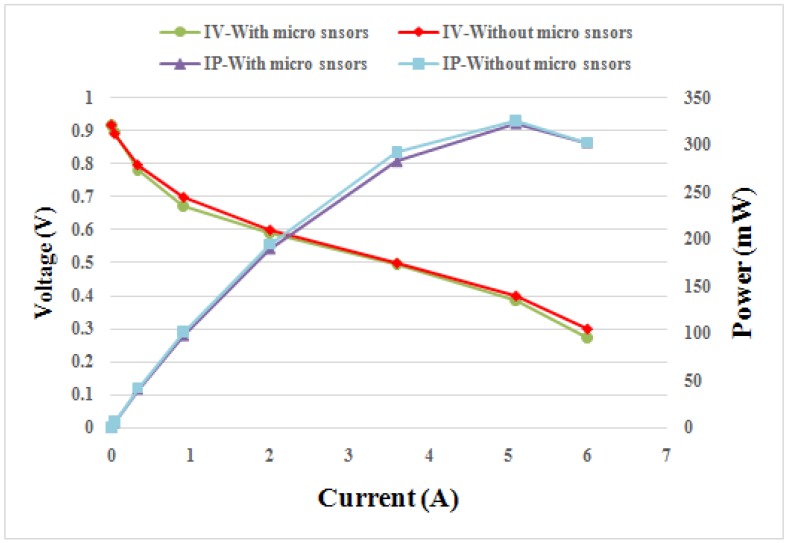
Comparison between performances of cells with and without embedded flexible integrated microsensor.

Figure 9 compares the performance curves of fuel cells with and without embedded flexible integrated microsensor at 50 °C. The flexible integrated microsensor embedded in the cell will cover the reaction area of MEA, preventing the gas from reacting with the covered MEA. However, the flexible integrated microsensor is very small; the area of three flexible integrated microsensors is about 2% of the overall reaction area of MEA. According to Table 2, the effect of the flexible integrated microsensor embedded in the cell on the performance is about 1%.

### 4.3. Local Temperature Distributions of Wired and Wireless Sensors

**Figure 10:**
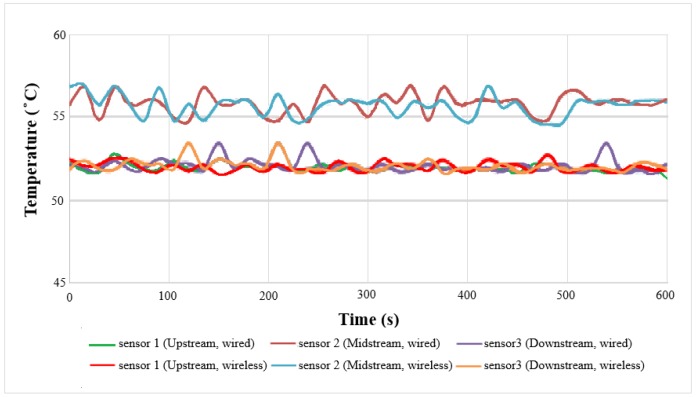
Local temperature distributions of wired and wireless sensors.

The local temperature distributions of wired and wireless measurement at 50 °C and low current (2 A) are shown in Figure 10. The midstream temperature is higher than upstream and downstream temperatures, the maximum value is 56.8 °C, because the midstream is located in the center of the cell, where there is a hot stack. The upstream and downstream temperatures are lower than the midstream temperature, because the flow carries more heat away at the gas inlet and outlet. The minimum value is 52.1 °C. The overall temperature is higher than the outside: 50 °C. As the microsensor is in the cell, it can measure the actual internal situation, the outside is affected by room temperature, the internal temperature is higher than the external temperature.

### 4.4. Flow Distributions of External, Wired and Wireless Sensors

**Figure 11:**
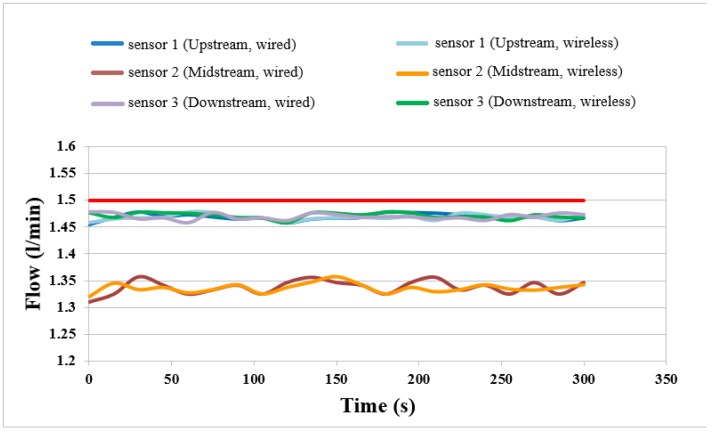
Flow distributions of external, wired and wireless sensors.

The flows of external, wired and wireless sensors at constant current (2 A) and temperature 50 °C are compared in Figure 11. It is observed that the upstream and downstream flows are higher than the midstream flow. This may be because the upstream flow is near the gas inlet. The gas is sufficient. The midstream flow is lower because the cell is provided with serpentine for better performance. The gas distribution in the middle reach is nonuniform. The wireless and wired measurements are compared. The variation curve trends are consistent, validating the feasibility of the wireless flow sensing.

### 4.5. Local CO Concentrations of Wired and Wireless Sensors

**Figure 12:**
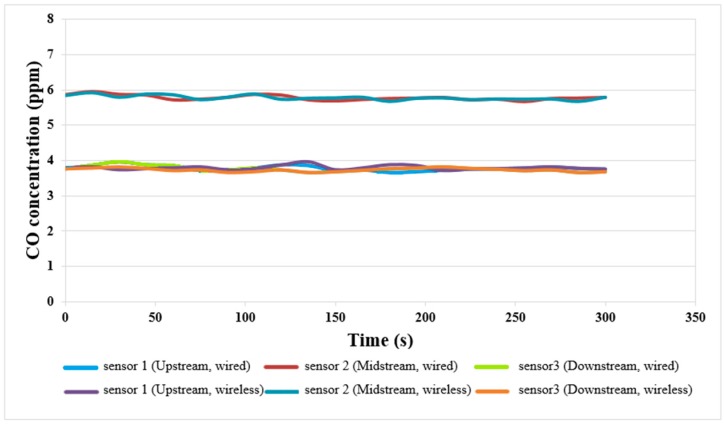
Local CO concentration distribution diagram of wired and wireless sensors.

The defect in the fuel cell is incomplete combustion, the catalyst Pt is poisoned by CO, so that the cell performance is degraded. At present, the external measurement cannot obtain the CO volume in the cell, and the external invasion is likely to result in an air leak. Therefore, this study uses a micro CO sensor for non-invasive internal measurement. The local CO distributions of wired and wireless sensors are shown in Figure 12. As the measured value is lower than the range of the correction value, the linearity between the measured values 0 ppm and 10 ppm is adjusted. The upstream and downstream have lower CO concentration. This may be because the upstream and downstream are located at the gas inlet and outlet. The charge of fuel is large and the CO concentration is relatively lower; the minimum value is 3.69 ppm. The midstream has less charge of fuel and relatively higher CO concentration; the maximum value is 5.96 ppm.

## 5. Conclusions

This study uses MEMS technology to develop an electrochemical environment resistant flexible integrated (temperature, flow and CO) microsensor for 50 μm thick PI substrate. The protection layer is made of PI (PI 7320) with good temperature tolerance. Three electrochemical environment resistant flexible integrated microsensors are embedded in the upstream, midstream and downstream of the cathode channel plate of a fuel cell without affecting the operation of PEMFC. The temperature, flow and CO correction curves of flexible integrated microsensor are measured. The results show that the linearity and reliability are good. The comparison results of the performance curves of cells with and without embedded flexible integrated microsensor show that the flexible integrated microsensor embedded in the cell influences about 1% of performance.

In addition, the local temperature, flow and CO in the fuel cell are monitored by wired and wireless sensors. According to the test at the operating temperature of 50 °C and constant current (2, 3.5, 6 A), the curves of local temperature, flow and CO in the cell have consistent trends in wired and wireless monitoring, validating the reliability of a wireless flexible integrated microsensor. This study provides the internal information of the cell for reference, so that three important physical parameters in the fuel cell (temperature, flow and CO) can be known and provided to designers and operators in order for them to make improvements and adjustments.

## Figures and Tables

**Table 1:** Fuel cell test conditions.

Item	Condition
Cell temperature (°C)	50
Anode terminal flow (H_2_) (slpm)	0.5
Cathode terminal flow (Air) (slpm)	1.5
Gas temperature (°C)	Room temperature
Constant current (A)	2, 3.5, 6
Reaction area (cm^2^)	6.25

**Table 2:** Reaction area and maximum power of cells with and without embedded flexible integrated microsensor.

		With Integrated Microsensor	Without Integrated Microsensor
	
Reaction area (cm^2^)		6.125	6.25
Maximum power (mW)		323.56	326.9
